# Supplementary material for: Stereotactic Radiofrequency Ablation for Treatment-Refractory Depression: A Systematic Review and Meta-Analysis
Source: Brain Sci. 2022 Oct 12;12(10):1379. doi: 10.3390/brainsci12101379 (PMC9599878; doi:10.3390/brainsci12101379)
Supplement: Supplementary file 1 [file brainsci-12-01379-s001.zip › brainsci-1952224-supplementary.pdf]

Online Supplementary material

***Table S1*: Typical inclusion and exclusion criteria for NMD.**
The listed criteria are merged from the reported studies and adapted from Hurwitz et al.

Inclusion Criteria	Exclusion Criteria
Age >18-20 years or older.	Age <18 years.
No sufficient benefit from adequate periods of treatment including pharmacotherapy, augmentation strategies and ECT.	Failure to satisfy the inclusion criteria.
Duration of illness >3 years, with at least 2-5 years of unremitting symptoms despite treatment.	A diagnosis of organic brain syndrome including Alzheimer's Disease, vascular and other dementias, atrophy, or tumour.
Assessment by a multidisciplinary team including a psychiatrist, neuropsychologist and neurosurgeon who approve that the patient meets the criteria of TRD.	A current diagnosis of substance misuse including alcoholism or mental and behavioural disorders due to psychoactive substance use.
Freely given and fully informed consent confirmed by an independent assessor (e.g., the Care Quality Commission in the UK), who confirm that the surgery is appropriate.	A significant psychiatric or physical comorbidity including a diagnosis of a personality, delusional, organic mental, or pervasive developmental disorder.
Written informed consent provided by the patient and the agreement to participate in the preoperative evaluation program and postoperative rehabilitation program.	Absolute contraindications to neurosurgery (e.g., life-long anticoagulants).
The referring psychiatrist has to accept the responsibility for the postoperative long-term management of the patient. *	

* This criterion is not the preferred model anymore. Today, specialist psychiatric services have a shared-care plan with local services.

***Table S2*: Overview of patient response rates.**

Study	N	Responder	Partial-Responder	Improvement	Deteriorated
**ACING**					
J. D. Steele, et al. 2008. Biol Psychiatry; 63: 670-677.	5	60%	20%	20%	0%
**ACAPS**					
D. Christmas, et al. 2011. J Neurol Neurosurg Psychiat; 82: 594-600.	11	36%	0%	46%	18%
L. Subramanian, et al. 2017. Psychol Med; 47: 1097-1106.	21	57%	10%	24%	9%
J. M. Avecillas-Chasin, et al. 2019. Stereotact Funct Neurosurg; 97: 369-380.	10	60%	20%	10%	10%
**Total**	47	53%	11%	26%	10%

***Table S3*: Overview of patient data where there are complete pre- and post-surgical outcomes reported on the same scale**

**Study**	**Patients treated**	**Patients reported**	**Individual patient data available (Y/N)**	**Percentage of data complete**
**ACING**				
J. D. Steele, et al. 2008. Biol Psychiatry; 63: 670-677.	8	5	Y	62.5%
D. C. Shields, et al. 2008. Biol Psychiatry; 64: 449-454.	33	17	Y	51.5%
**ACAPS**				
D. Christmas, et al. 2011. J Neurol Neurosurg Psychiat; 82: 594-600.	20	11	Y	55.0%
L. Subramanian, et al. 2017. Psychol Med; 47: 1097-1106.	45	21	Y	46.6%
J. M. Avecillas-Chasin, et al. 2019. Stereotact Funct Neurosurg; 97: 369-380.	10	10	Y	100%
